# Cancer awareness among community pharmacist: a systematic review

**DOI:** 10.1186/s12885-018-4195-y

**Published:** 2018-03-16

**Authors:** Kofi Boamah Mensah, Frasia Oosthuizen, Adwoa Bemah Bonsu

**Affiliations:** 1 National Center for Radiotherapy & Nuclear Medicine, Directorate of Oncology, Komfo Anokye Teaching Hospital, Box 1934, Kumasi, Ghana; 2 University of KwaZulu-Natal, Discipline of Pharmaceutical Sciences, College of Health Sciences, Westville Campus, University Road, Durban, South Africa; 3 Kwame Nkrumah University of Science & Technology, Department of Nursing, College of Health Sciences, Kwame Nkrumah University of Science & Technology, Kumasi, Ghana

**Keywords:** Cancer, Awareness, Signs and symptoms, Screening, Community pharmacists

## Abstract

**Background:**

The WHO recognises that community pharmacists are the most accessible healthcare professionals to the general public. Most patients regularly visit community pharmacies for health information and also seek advice from pharmacists with respect to signs and symptoms of cancer. As readily accessible health care professionals, community pharmacists are also in the best position to include cancer-screening initiatives into their practice. Pharmacists are therefore in a good position to raise awareness when they counsel people who buy over-the-counter medication for the control of possible cancer-related symptoms. The aim of this review was to critically appraise evidence gathered from studies that; (1) explore or assess knowledge of community pharmacist on signs and symptoms of cancer, (2) explore or assess knowledge of community pharmacist on cancer screening.

**Methods:**

EMBASE (ovid), CINAHL (EBSCOhost) and MEDLINE (EBSCOhost) were systematically searched for studies conducted between 2005 to July 2017. Studies that focused on knowledge of community pharmacist in cancer screening, signs and symptoms were included.

**Results:**

A total of 1538 articles were identified from the search, of which 4 out of the 28 potentially relevant abstracts were included in the review. Findings of the selected studies revealed lack of sufficient knowledge on breast cancer screening, signs and symptoms. Both studies attributed knowledge limitation as the cause of reason for the key findings of their studies.

**Conclusion:**

The selected studies focused largely on breast cancer, which hinder the generalizability and transferability of the findings. Hence there is a need for more studies to be conducted in this area to draw a better conclusion.

## Background

Cancer now causes more deaths than all coronary heart disease or strokes, according to World Health Organisation (WHO) estimates for 2011 [27]. This is likely as a result of late presentation of the disease [16] which have been attributed to a number of factors such as poor awareness of the signs and symptoms of cancer, cancer risk factors, poor availability of tests or screening programs [9, 12, 13].

The continuous global demographic and epidemiological evolution shows an increasing cancer burden over the next decades, especially in low and middle income countries (LMIC), with over 20 million new cancer cases expected annually as early as 2025 [5].

Contemporary pharmacy practice reflects an emerging paradigm from one in which the pharmacist primarily supervises medication distribution and counsels patients, to a more expanded role providing patient-centered medication therapy management, health improvement, health education, health promotion activities and disease prevention services [25]. The role of the pharmacist in cancer care is now growing with community pharmacists advocating, promoting, supporting and providing cancer related health promotion [6].

The WHO recognises that community pharmacists are the most accessible healthcare professionals to the general public [1]. Studies have shown that community pharmacies provide easy and equitable access to healthcare [22]. Most patients regularly visit community pharmacies for health information and also seek advice from pharmacists with respect to signs and symptoms of cancer [15]. Pharmacists are therefore in a good position to raise awareness when they counsel people who buy over-the-counter medication for the control of possible cancer-related symptoms. To be able to achieve this, as healthcare providers in the community, pharmacist must be able to differentiate between conditions that require self-medication and those that need the attention of a physician. They must be able to identify the common signs and symptoms of cancer. As readily accessible health care professionals, community pharmacists are in the best position to include cancer-screening initiatives into their practice. A number of organizations including the US Preventive Services Task Force [24], American Cancer Society [21], and National Comprehensive Cancer Network [17], have developed cancer screening recommendations. Because clinicians may use different guidelines, pharmacists need a working knowledge of basic recommendations [20].

Studies that have assessed knowledge on screening, signs and symptoms of cancer among community pharmacist have been conducted, however, no systematic review have been conducted to pool findings from these studies to inform practice. The aim of this review was to critically appraise evidence gathered from studies that; (1) explore or assess knowledge of community pharmacist on signs and symptoms of cancer, (2) explore or assess knowledge of community pharmacist on cancer screening [23].

## Methods

### Sources and search strategy

Search of EMBASE (ovid), CINAHL (EBSCOhost) and MEDLINE (EBSCOhost) were done to identify evidence. The search period was from 2005 to July, 2017. The MEDLINE search strategy (Appendix 1) used key words such as cancer, community pharmacist, knowledge, awareness, signs and symptoms, screening. This search strategy was adopted for other databases search. Additional search from reference lists of articles selected for full text review yielded no results. The review was designed and carried out following established guidelines on good conduct and reporting of systematic reviews [14]. The protocol was registered with PROSPERO [23], registration number 2017:CRD42017071390.

### Eligibility criteria and study selection

**Fig. 1:**
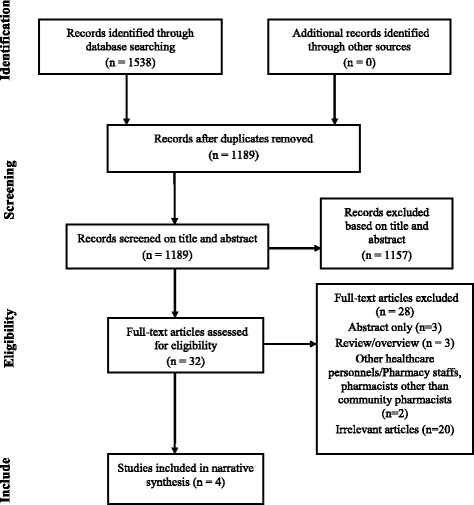
Systematic selection process

Two investigators (KM and FO) independently read the titles and abstracts of all records retrieved and assessed them against the set criteria (Table 1). Data from the included studies were extracted by the primary reviewer (KM) using a standardized research matrix [10], and later checked by another reviewer (AB). Author’s name, year of publication, country and setting, study design, type of cancer, sample size, findings, where the data collected (Appendix 2). The search results were independently reviewed by two authors (KM and FO). The database search identified 1538 records. A total of 349 duplicate records were deleted. One thousand one hundred and eight nine (1189) articles were independently screened on title and abstract by two authors (KM and FO) and irrelevant articles were excluded. The authors evaluated 32 full-text articles for eligibility. After exclusion of 28 articles, 4 studies met the criteria for inclusion in the review. A flow chart summarising the selection procedure is shown in Fig. 1.

**Table 1:** Inclusion and exclusion criteria

Inclusion criteria	Exclusion criteria
➢ Study population includes community pharmacists. ➢ All study types on signs and symptoms of cancer ➢ All study types of cancer screening. ➢ All study design. ➢ Studies published from 2005 to July, 2017 ➢ Full text available ➢ Abstract available ➢ Studies published in English language	➢ Studies not related to cancer signs and symptoms. (Irrelevant articles) ➢ Studies not related to cancer screening. (Irrelevant articles) ➢ Studies related to pharmacy staffs, pharmacists, other healthcare personnels other than community pharmacists ➢ Abstract ➢ Conference abstract ➢ Overview/ review ➢ Studies with full text not available in English language. ➢ Studies published before 2005

## Results

The database search found 1538 publications between 2005 and July 2017. A total of 349 duplicate records were removed. A further 1173 records were excluded based on their abstracts and titles. Following the exclusion criteria, another 28 records were also excluded. The remaining 4 articles which met the inclusion criteria were read in full. A flowchart summarising the selection process is shown in Fig. 1.

### Study characteristics

The characteristics of the four studies are shown in Table 2. The studies were published from 2010 to 2016. The studies were conducted in Malaysia, Qatar, UAE and Jordan. Community pharmacists were recruited from commercial community pharmacies. The studies included a total of 1678 pharmacists. Breast cancer was the type of cancer discussed in the selected studies. The smallest sample size in the studies was 35 [3] and largest sample size was 1113 [2].

**Table 2:** Characteristic of studies included in the review

Lead author	Year	Country	Sample description	Type of cancer	Duration of Study	Study design	Sample size	Conclusion
Ibrahim	2013	UAE	Community pharmacies	Breast	24 weeks	Cross sectional survey	335	Community Pharmacists have low level of knowledge in breast cancer. Efforts should be made on providing pharmacists with high quality Breast cancer continuous education.
Beshir	2012	Malaysia	Independent community pharmacies	Breast	20 weeks	Cross sectional survey	35	Community pharmacists have low breast cancer knowledge which can prevent actualisation of pharmacist role in breast cancer education. Therefore further work should focus on providing pharmacists with high quality breast cancer continuous education.
EL Hajj	2011	Qatar	Community pharmacies	Breast	12 weeks	Descriptive cross sectional survey	195	Low breast cancer knowledge was recorded among community pharmacists. Further work should focus on providing pharmacists with breast cancer continuous education.
Ayoub	2016	Jordan	Commercial community pharmacies	Breast	20 weeks	Descriptive cross sectional survey	1113	There is knowledge gap pertaining to breast cancer and screening guidelines. Pharmacist must improve their knowledge through better undergraduate oncology education and intensive continuous education programmes

**Table 3:** Quality Assessment of Selected Studies

Study	Quality Assessment items					Relevance to Current Review		Score (%)
	A	B	C	D	E	F	G	
[3]	1	1	1	1	1	1	1	100
[8]	1	1	1	1	1	1	1	100
[11]	1	1	1	1	1	1	1	100
[2]	1	1	1	1	1	1	1	100

Total score divided by the total number of items multiplied by 100

0 = No or not reported; 1 = Yes;

A—was sample likely to be representative of the study population?, B—Was a response rate mentioned within the study?, C—Was the instrument used reliable?, D—Was the instrument used valid?; E—Was it a primary data source?; F—Was knowledge on signs and symptoms of cancer assessed?; G—Was knowledge on cancer screening assessed?

Quality assessment score matched with the objectives of the selected studies review: weak: 0–33.9%, moderate: 34%–66.9%, strong: 67%–100%

### Quality assessment of selected studies

The quality of the selected studies was assessed using a quality assessment tool [19] Score from 0% - 33.9% is regarded as weak, 34% - 66.9% is regarded as moderate, and 67% - 100% is regarded as strong (*n* = 4) based on [18] classification of quality level (Table 3).

## Discussion

The data from the selected studies were heterogeneous; hence it was not possible to combine it for meta-analysis. Hence the outcomes of the studies were reported as a narrative synthesis.

Findings of the four selected studies revealed lack of sufficient knowledge on breast cancer and screening recommendations. Scores of participants on items about knowledge on cancer signs and symptoms were moderate ([2, 11]. The other two studies [3, 7] had only one item on cancer signs and symptoms which does not give a proper reflection about participants knowledge on signs and symptoms about breast cancer. With aging population in the world, the global burden of cancer is set to increase [4]. One of the approaches adopted by the World Health Organisation (WHO) is to raise awareness through education regarding warning signs of cancer [26]. Therefore much has to be done to improve the knowledge of community pharmacist on these warning signs. Scores were noticed to be low for items about knowledge on cancer screening recommendations for one of the studies [11]. All studies attributed knowledge limitation as the cause of reason for the key findings of their studies. Lack of continuous pharmacy education, non-attendance of continuous pharmacy education and different undergraduate pharmacy curricula contribute to knowledge limitations.

Through this systematic review it can be seen that there has not been many studies done to analyse the knowledge of community pharmacist on screening recommendations, signs and symptoms of cancer for the past 12 years. The selected studies focused on breast cancer only, which hinder the generalizability and transferability of the findings. Hence there is a need for more studies to be conducted in this area to draw a better conclusion which will inform policy.

## Conclusion

In conclusion, community Pharmacists possess moderate knowledge on breast cancer signs, symptoms and screening recommendations. However, the findings of this systematic review were highly limited by the fact that only four studies met the review criteria, samples of studies were taken from only one geographic area, Middle East Region and sample size was relatively small. Hence findings may not be applicable to all community pharmacists in general. Further studies should be conducted in other sub regions of the World to generate results for future policy implementation.

### Limitations

The search was limited to three databases and did not include data from grey literature. Also the search was restricted to studies conducted from 2005 to July, 2017 and studies published in English. These create opportunity for study selection bias.

Researcher–designed questionnaires were used in the selected studies, which led to heterogeneous results that could not be combined for meta-analysis or meta-synthesis.

The studies were done in only breast cancer hence cannot be generalised for the other cancers. The review was limited to four studies only, and so worldwide survey is required to address certain perception aspects of breast cancer screening, signs and symptoms.

## Appendix 1

### An example of search strategy used in the Medline Ovid platform.

1. ‘cancer* OR ‘tumour * OR ‘tumor* ‘OR ‘neoplasm* OR ‘malignant* OR ‘neoplasm malignant* OR ‘malignant neoplasm* OR ‘tumoral disease* OR malignant tumor* OR ‘malignant tumour* OR ‘cancer morphology’ OR ‘malignant tumor morphology’ OR ‘primary malignant neoplasm*
2. ‘community pharmacist* OR community pharma* OR ‘community druggist* OR ‘pharmacist*
3. ‘know* OR ‘knowledge’ OR ‘knowledges’
4. ‘awareness OR ‘awarenesses’ OR aware*
5. ‘signs and symptoms’ OR ‘sign* OR ‘symptom*
6. screening* OR ‘early detection of cancer’
7. #1 AND #2
8. #3 OR #4
9. #5 OR #6
10. #8 OR #9
11. #7 AND #10

## Appendix 2

**Table 4:** A summary of the findings of studies that were reviewed

Title	Year	Country	Site	Sample size	Findings	Limitation
Breast cancer health promotion in Qatar: a survey of community pharmacists’ interests and needs.	2011	Qatar	Community pharmacies	195	• Breast cancer knowledge was evaluated using twelve true or false breast cancer related questions. • Eighty-eight respondents (48%) scored less than 60% and only 21 respondents (11%) scored more than 80%. The mean percent score was 63 ± 15%. • One hundred and forty respondents (77%) expressed high interest in receiving breast cancer continuous education. • Community pharmacists’ perceived barriers for providing breast cancer health promotion. Highly perceived barriers included lack of breast cancer educational materials (79% of respondents), lack of personnel (59%), and lack of public recognition of this pharmacist’s role (61%) and lack of time (51%).	• As this was a self-reported survey, the responses may have contained some data inaccuracies resulting from intentional deception, poor recall of information, or misunderstanding of the question and may be biased by an inclination to provide social desirable responses and acquiescence. • The survey reliability was not tested among the population of Qatar’s community pharmacists. • The survey was only completed by 60% of community pharmacists in Qatar. Thus generalization of the study results to all Qatar’s pharmacists should be made carefully.
Knowledge, Perception, Practice and Barriers of Breast Cancer Health Promotion Activities among Community Pharmacists in Two Districts of Selangor State, Malaysia.	2012	Malaysia	Independent community pharmacies	35	• Breast cancer knowledge was evaluated using 7 questions to be answered as yes, no or uncertain. More than half (50%) of the participants answered general questions related to breast cancer and its risk factors correctly. The mean percent score of correct answers was 72.6% ± 11. • Pharmacists’ perception regarding breast cancer health promotion was assessed using 8 lickert scale type questions. The mean percent score for the pharmacists who strongly agree or agree with all the given statements was 90% ± 8.07. • 91.4% strongly agree or agree that there is a need to integrate breast cancer health promotion activities i to their daily practice and about 71.4% strongly agree or agree that there is a demand from the community to get advice on breast cancer screening and early detection. • The community pharmacists cite lack of time (80%), lack of breast cancer education materials (77.1%), and training (62.9%) as major barriers that limit their involvement in breast cancer promotion activities.	• The cross-sectional survey was confined to community pharmacists in two districts (Malaysia), hence the results could not be generalized to all community pharmacists in Malaysia.
Community Pharmacists’ involvement in Breast Cancer Health Promotion in United Arab Emirate (UAE).	2013	UAE	Community pharmacies	335	• About 47% of the pharmacists reported that they never provided patients with advice or counselling on breast cancer screening and early detection. • 67% never provided patients with breast cancer educational materials or self-assessment. • 96% of them never invited healthcare professionals to provide breast cancer education to patients in the pharmacy. • 75% indicated that they were highly interested in providing breast cancer health promotion and 162 respondents (59%) were highly comfortable in delivering this activity. • 78% were highly interested in receiving breast cancer continuous education. • 65% answer correctly question on cancer sign, 13% answer incorrect and 22% did not know the answer. • Scores were noticed to be low for questions associated with breast cancer screening recommendations and risk factor. • 87% of the participants believed that discussing breast cancer awareness with female patients in the pharmacy is beneficial and can save their lives. • 86% agreed that their professional status and satisfaction can be improved through provision of breast cancer counselling in the pharmacy. • Highly identified barriers included respectively: deficiency in breast cancer educational materials (87% of participants), lack of time (74% of participants), insufficient personnel (68% of respondents) and lack of reimbursement for such services (50% of participants).	• The study variables were assessed by self-report, which may be biased by an inclination to provide socially desirable responses, acquiescence (tendency to agree) and extremity (tendency to use extreme ratings). • The survey reliability was not tested among the population of UAE’s community pharmacists. • The survey sample size was relatively small.
Knowledge, Attitudes and Barriers towards Breast Cancer Health Education among Community Pharmacists.	2016	Jordan	Commercial community pharmacies	1113	• 56.7% agreed to receive adequate education, while 30.5% disagreed and 12.8% provided a neutral response. • 54.9% admitted their information about oral chemotherapeutic agents were gained primarily through their work as community pharmacists. • Majority of community pharmacists reported no attendance of continuous education activities in relation to oncology (63.4%) or breast cancer (62.3%) during the last 2 years. • A small percentage of surveyed pharmacists reported attending more than two continuous educational activities related to oncology (4.9%) or breast cancer (4.3%) over the past 2 years. • (81.5%) agreed to the fact that breast cancer is the most commonly diagnosed type of cancer among women worldwide. • When asked if breast cancer should not be of concern for patient younger than 40 years, 54.9% of participants agreed that this statement was incorrect. • Regarding the initial signs and symptoms of breast cancer, majority of pharmacists (70.3%) agreed that a painless lump is the initial sign. • Enquiring about findings in advanced breast cancer (54.3%) of respondents agreed that pain, nipple discharge and skin oedema are common findings in this stage. • (6.7%) considered breast feeding a risk factor for breast cancer development. • (81.8%) reported family history as a leading factor for breast cancer development. • Overall assessment of pharmacists’ knowledge revealed that half the pharmacists (50%) had poor knowledge, while the other half had acceptable level of knowledge of breast cancer. • only nine pharmacists (0.9%) had a total score of 15 points on assessment of breast cancer knowledge. • 64%) agreed that counselling women about BSE should start at age of 30 which was incorrect. • With respect to frequency of BSE, large proportion of respondents (70.7%) answered correctly to recommend once monthly BSE examination. • The overall mean score for pharmacists’ knowledge of screening guidelines was 3.83 ± 1.61 out of a maximum score of 7 points (median = 4, range 0–7) classifying the knowledge as satisfactory. • 60% had poor knowledge, while 40% had satisfactory knowledge of screening recommendations. • (3%) had a total score of 7 points (100%) based on knowledge of breast cancer screening • 46.5%) strongly agreed that pharmacists should be involved in breast cancer health promotion in community pharmacy settings. • Mean score for pharmacist attitude was 19.82 4.34 out of a maximum score of 28 points (median = 21, range 6–28), • 89.7% had favourable attitude compared with 103 pharmacists (10.3%) who were classified as having less favourable attitude. • Recognised barriers were lack of privacy (57.1%), lack of skills (56.2%) and lack of adequate knowledge (50%). • 20.2% reported lack of direct profit as a barrier to active involvement in patient education.	• Self-reported design used in the study may have contained some data inaccuracies and may not accurately reflect what pharmacists actually do in practice. • The convenience sample which was used may create a selection bias which limits the generalizability of results.

## Abbreviations

LMIC: Low and middle income countries
WHO: World Health Organisation
